# Identification of genetic loci affecting body mass index through interaction with multiple environmental factors using structured linear mixed model

**DOI:** 10.1038/s41598-021-83684-1

**Published:** 2021-03-02

**Authors:** Hae-Un Jung, Won Jun Lee, Tae-Woong Ha, Ji-One Kang, Jihye Kim, Mi Kyung Kim, Sungho Won, Taesung Park, Ji Eun Lim, Bermseok Oh

**Affiliations:** 1 Department of Biomedical Science, School of Medicine, Kyung Hee University, Seoul, South Korea; 2 Department of Biochemistry and Molecular Biology, School of Medicine, Kyung Hee University, Seoul, South Korea; 3 Department of Preventive Medicine, College of Medicine, Hanyang University, Seoul, South Korea; 4 Department of Public Health Science, College of Medicine, Hanyang University, Seoul, South Korea; 5 Department of Public Health Science, Seoul National University, Seoul, South Korea; 6 Department of Statistics, Seoul National University, Seoul, South Korea

**Keywords:** Computational biology and bioinformatics, Genetics

## Abstract

Multiple environmental factors could interact with a single genetic factor to affect disease phenotypes. We used Struct-LMM to identify genetic variants that interacted with environmental factors related to body mass index (BMI) using data from the Korea Association Resource. The following factors were investigated: alcohol consumption, education, physical activity metabolic equivalent of task (PAMET), income, total calorie intake, protein intake, carbohydrate intake, and smoking status. Initial analysis identified 7 potential single nucleotide polymorphisms (SNPs) that interacted with the environmental factors (*P* value < 5.00 × 10^−6^). Of the 8 environmental factors, PAMET score was excluded for further analysis since it had an average Bayes Factor (BF) value < 1 (BF = 0.88). Interaction analysis using 7 environmental factors identified 11 SNPs (*P* value < 5.00 × 10^−6^). Of these, rs2391331 had the most significant interaction (*P* value = 7.27 × 10^−9^) and was located within the intron of *EFNB2* (Chr 13). In addition, the gene-based genome-wide association study verified *EFNB2* gene significantly interacting with 7 environmental factors (*P* value = 5.03 × 10^−10^). BF analysis indicated that most environmental factors, except carbohydrate intake, contributed to the interaction of rs2391331 on BMI. Although the replication of the results in other cohorts is warranted, these findings proved the usefulness of Struct-LMM to identify the gene–environment interaction affecting disease.

## Introduction

Gene-environment interaction (GEI) studies evaluate the extent to which the phenotype was affected by the interaction^1^. These studies help in our understanding of the complex human traits that are determined through the interaction of gene and environment^2^. In addition, the gene identified through this analysis can help in discovering the biological pathways underlying the phenotype^3^ and in increasing the accuracy of prediction of the disease incidence, calculated by the main genetic and environmental effects^2,4^. Therefore, series of genome-wide interaction studies (GWIS) have been progressed to identify the genetic loci interacted with environmental factors in diverse traits^5–7^.

Obesity is a serious environment-related disease that affects many people across the world. Severely obese people have been reported to die 8 to 10 years earlier than those with normal weight. Moreover, obesity is strongly associated with diseases such as cardiovascular diseases, type 2 diabetes, and cancer. In the past 2 decades, the global incident rate of obesity has rapidly increased, and the prevalence of obesity is estimated to double in the next decade^8^. Studies have demonstrated that body mass index (BMI), an index for obesity, is influenced by environments such as physical activity, nutrient intake, and diverse environmental exposure. Likewise, it is well known that BMI is affected by multiple genetic factors, which have been identified by series of genome-wide association studies (GWAS)^9^. Among the GWAS single nucleotide polymorphisms (SNPs), the *FTO* locus is the strongest genetic variant and is recently reported to affect BMI through interaction with environmental factors, including physical activity, diet, and smoking^7,10–13^.

Most of the interaction studies for obesity calculate the interaction between a single genetic factor or multiple genetic factors and a single environment factor^9,14–17^. However, there is a possibility that the interaction of multiple environments and a single genetic factor can also affect the phenotype^7,18^. Recently, the structured linear mixed model (Struct-LMM) analysis was reported to identify the genetic loci, characterized by the interaction of multiple environmental factors^18^. Using this method, high-dimensional environmental data can be used in population cohorts to help understand the effects of genetic factors in a group on complex traits and diseases^18^.

In this study, we applied the Struct-LMM method to the Korea Association Resource (KARE), a well-known Korean population GWAS database, comprised of 8,840 participants^19^. A total of 8 obesity-related environmental factors, which could interact with multiple environmental factors for BMI, were examined on genome-wide genetic variants. These environments included estimated daily alcohol consumption, education, physical activity metabolic equivalent of task (PAMET), income, total calorie intake, protein intake, carbohydrate intake, and smoking status.

## Materials and methods

### Korean association resource (KARE) cohort

**Figure 1:**
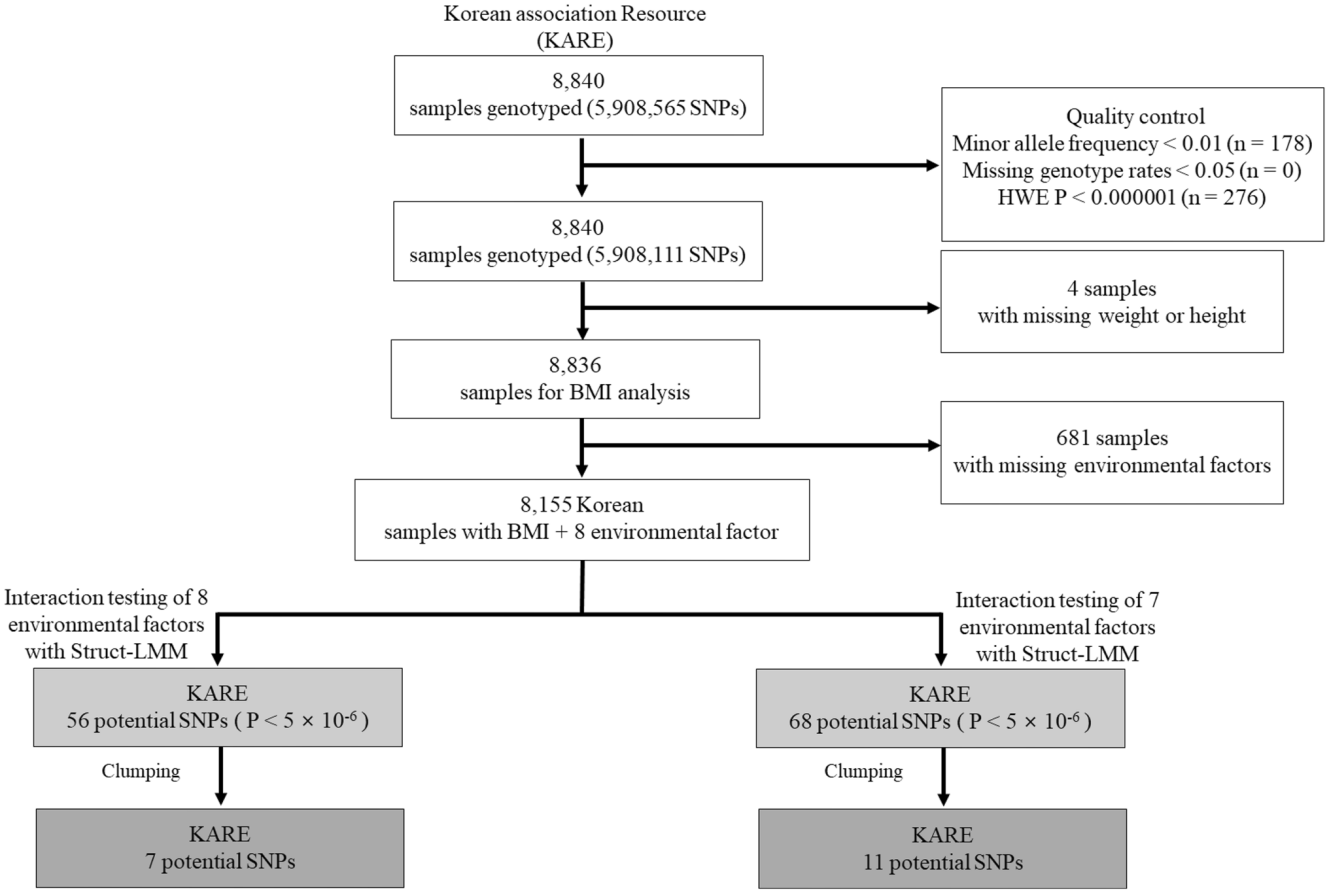
Diagram showing the study design. Left side of the diagram depicts the interaction between 8 environmental factors and 5,908,111 single nucleotide polymorphisms (SNPs), and the right side of the diagram depicts the interaction between 7 environmental factors (without physical activity) and 5,908,111 SNPs. The numbers in the parenthesis indicate the SNPs excluded by the specified quality control. *BMI* body mass index.

We utilized data from KARE for all the analyses. Participants of KARE cohort were recruited from two regions in South Korea (Ansan and Ansung) from 2009 to 2012 for the Korean Genome and Epidemiology Study. All study participants aged ≥ 40 years provided written informed consent, and approval was obtained from the institutional review board. The exclusion criteria were as follows: history of cancer, gender inconsistencies, cryptic relatedness, low genotype call rate (< 95%), and sample contamination (Fig. 1)^19,20^.

From the 8,840 samples, 8,155 samples (with all the 8 environmental factors related to obesity) were selected. The distribution of the variables in the samples has been summarized in Table 1.

**Table 1:** Basic characteristics of Korean Association Resource (KARE) participants included in this study, stratified by weight as a criterion. The numbers with percentile indicate mean and standard deviation (SD), and the numbers without percentile indicate the sample number in the variable.

KARE cohort	Underweight	Normal weight	Overweight	Obese
	BMI < 18.5	(18.5 ≤ BMI < 23)	(23 ≤ BMI < 25)	(BMI ≥ 25)
Number of participants	146	2347	2138	3524
Males (%)	60.96%	51.34%	47.99%	47.21%
age (mean, SD)	56.35(9.74)	52.23(9.38)	51.56(8.73)	52.10(8.54)
Height (cm) (mean, SD)	160.66(7.73)	160.62(8.53)	160.33(8.47)	159.69(8.94)
Weight (kg) (mean, SD)	45.60(5.29)	55.29(6.55)	61.91(6.70)	70.06(8.88)
Body mass index (kg/m^2^) (mean, SD)	17.60(0.82)	21.37(1.16)	24.02(0.57)	27.42(2.07)
For estimated day consumption of alcohol (g/day) (mean, SD)	1.84(1.27)	1.78(1.13)	1.79(1.10)	1.78(1.13)
**Education**				
Less than primary school	71	736	637	1214
Completed middle school	28	530	470	796
Completed high school	30	781	724	1020
Completed college	5	90	80	116
Completed university	12	183	195	319
More than university	0	27	32	59
PAMET (mean, SD)	11,288.22(7820.73)	10,029.26(6481.24)	9519.96(6069.51)	9271.68(6038.71)
**Income**				
$\times$ < 500,000 won	55	465	349	611
500,000 won ≤ $\times$ < 1,000,000 won	23	396	340	504
1,000,000 won ≤ $\times$ < 1,500,000 won	21	345	318	548
1,500,000 won ≤$\times$x < 2,000,000 won	18	333	310	479
2,000,000 won ≤ $\times$ < 3,000,000 won	17	446	382	652
3,000,000 won ≤ $\times$ < 4,000,000 won	7	226	251	365
4,000,000 won ≤ $\times$ < 6,000,000 won	4	102	144	222
6,000,000 won < $\times$	1	34	44	93
Total calorie intake (kcal) (mean, SD)	1866.09(739.49)	1925.13(686.87)	1940.74(687.59)	1975.49(690.45)
Protein intake (g) (mean, SD)	61.75(30.15)	65.12(31.18)	66.49(27.62)	67.66(29.29)
Carbohydrate intake (g) (mean, SD)	332.10(134.90)	339.01(111.35)	340.36(119.58)	347.41(115.20)
**Smoking status**				
Never	62	1294	1247	2178
Previous	22	340	343	588
Sometimes	10	64	51	99
Often	10	649	470	659

### Genotype data

The KARE study utilized the Affymetrix Genome-Wide Human SNP Array GeneChip 5.0^19^. SNP imputation was performed using IMPUTE2 with the 1000 Genomes Project (haplotype phase 1)^19^. At the baseline, genetic data was available for 6,461,358 SNPs in samples of 8,840 KARE participants. We performed quality control based on the following exclusion criteria: variants with missing genotype call rates > 0.05, minor allele frequency < 0.01, and Hardy–Weinberg equilibrium *P* value < 1.00 × 10^−6^. Based on these criteria, 5,908,111 SNPs were included in the study (Fig. 1).

### Dataset for phenotype and environmental factors

The methods for the measurement of height and weight have been described in a previous study^20^. BMI was calculated as weight (kg)/height (m^2^). For analyses, BMI was transformed to normal distribution using Gaussian function in Struct-LMM.

The environmental factors used in Struct-LMM were selected based on previous studies on BMI^9,14,16,17^. The 8 environmental factors were alcohol consumption^9^, education^14^, physical activity^9^, income^9^, total calorie intake^16^, protein intake^16^, carbohydrate intake^16^, and smoking status^17^ (Supplementary Table 1).

The estimated daily consumption of alcohol (g/day) was calculated, as described previously^20^. PAMET was obtained from each participant using a structured questionnaire that included four types of physical activities including sleeping, five different sedentary activities, non-sedentary activities, and only leisure-time physical activity^21^. The total calorie intake, protein intake, and carbohydrate intake were calculated from a food frequency questionnaire (FFQ), as described^22,23^.

For the Struct-LMM interaction analysis of alcohol consumption, the amount of alcohol consumption additionally increased in each group by 20 g/day from the 1st group (less than 20 g/day) up to the 6th group (> 100 g/day)^24^. For education analysis, participants were divided into two groups, one with education no more than secondary school and another with education more than secondary school. The physical activity was analyzed from PAMET score as a continuous variable. For income analysis, participants were divided into groups based on various levels of income, as shown in Supplementary Table 1. For the analysis of total calorie intake, protein intake, and carbohydrate intake, participants were divided into four groups, as shown in Supplementary Table 1. For smoking analysis, participants were divided into two groups, one with no or previous history of smoking and the other with current smoking^17^.

### Statistical analysis

SNP quality control was performed in PLINK v.1.9.0, as described previously^25^. The gene-environment interaction on BMI for the eight environmental factors was analyzed by Struct-LMM v.0.3.1^18^, adjusted for age, sex, and recruitment area^26^.

We performed Manhattan plot drawing, box plot drawing, association analysis, residual value calculation, bar graph drawing, and correlation in R stats package version 3.5.1 (www.r-project.org). For Manhattan plot drawing, qqman package was used for residual value calculation, while for association analysis, lme4 package in R stats package was used. We calculated the Bayes Factor (BF) for each environmental factor to explore the most relevant environments for GEI in Struct-LMM v.0.3.1. The Bayes Factor method used in Struct-LMM is a statistical method comparing two models, one with the environmental factors and another without the environmental factors, in order to assess which model is better by quantifying the power of each model.

We performed a gene-based genome-wide association analysis using the MAGMA^27^ tool provided by FUMA^28^ through GWIS results calculated by Struct-LMM in this study.

### Ethics approval and consent to participate

This study was performed in accordance with the World Medical Association Declaration of Helsinki. All participants provided written informed consent to participate in the study^19^. Approval for the study was obtained from the Institutional Review Board (IRB) of Kyung Hee University (KHSIRB-19-387(EA), KHSIRB-20-077(EA)).

## Results

### Association of environmental factors on BMI

The environmental characteristics of the 8155 participants, included in this Struct-LMM analysis, have been summarized in Table 1. The participants were categorized into four obesity groups, based on the BMI values stated by the World Health Organization (Asia–Pacific region) and the Korean Obesity Society^29^. The average BMI of the participants was 24.61 (standard deviation = 3.12).

The relationship between the environmental factors was assessed using a correlogram (Supplementary Fig. 1). It was observed that PAMET and carbohydrate intake had relatively low correlation with other environmental factors, compared to the other 6 factors.

All the 8 environmental factors showed an association with BMI (*P*-value < 0.05). However, the associations of alcohol consumption (*P-*value = 2.83 × 10^−2^), education (*P*-value = 7.02 × 10^−3^), protein intake (*P*-value = 9.94 × 10^−3^), and carbohydrate intake (*P*-value = 1.02 × 10^−2^) with BMI, were not valid after the Bonferroni correction for multiple comparisons (*P*-value < 6.2 × 10^−3^) (Supplementary Table 1). Alcohol consumption, income, total calorie intake, and protein intake were positively correlated with BMI, while education, PAMET, carbohydrate intake, and smoking were negatively correlated with BMI. The order of effect size of the 8 environmental factors, regardless of positive or negative correlation with BMI, was as follows: protein intake classified as quartile (β = 0.81), alcohol consumption classified as 6 groups (β = 0.75), smoking status classified as smoker and non-smoker (β =  − 0.65), education classified as 2 groups (β =  − 0.21), total calorie intake classified as quartile (β = 0.15), carbohydrate intake classified as quartile (β = 0.08), income classified as 8 groups (β =  − 0.07), and PAMET as a continuous variable (β =  − 2.81 × 10^−5^) (Supplementary Table 1).

### Analysis of gene–environment interaction on BMI using Struct-LMM

**Figure 2:**
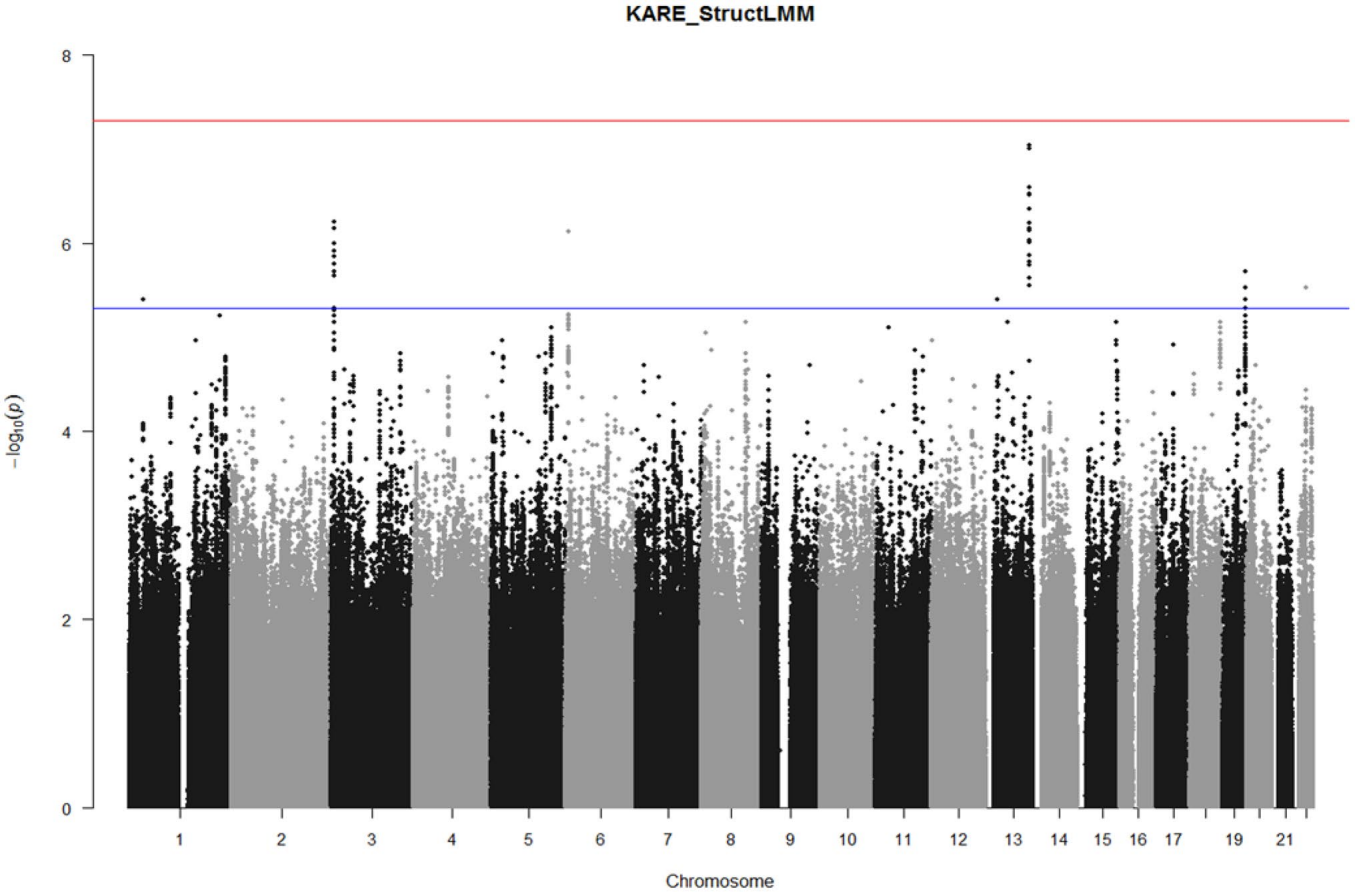
Manhattan plots for genome-wide interaction study using eight environmental factors. The red line indicates the suggestive threshold (*P* value < 5.00 × 10^−6^). The blue line indicates the genome-wide significance threshold (*P* value < 5.00 × 10^−8^).

We performed the GEI test between the 5,908,111 SNPs and 8 environmental factors on BMI, using Struct-LMM adjusted for age, sex, and recruitment area. The study design has been depicted in Fig. 1. Based on the results, we did not find an association of genome-wide significance with *P*-value < 5 × 10^−8^, as shown in the Manhattan plot (Fig. 2). However, we found 7 potential associations with *P*-value < 5 × 10^−6^ (Table 2).

**Table 2:** Interactions between individual single nucleotide polymorphisms (SNPs) and 8 environmental factors. ^a^Chromosomal positions are based on the 1000 Genomes Project’s haplotype phase 1 in NCBI build 37 (hg19). ^b^MAF, minor allele frequency. ^c^The *P* value for the effects of interaction between genotypes and environmental factors on BMI was assessed by using a Struct-LMM with adjustment for age, sex, and recruitment area.

Chromosome	Nearest gene	SNP ID	Position^a^	Minor allele	Major allele	MAF^b^ (%)	*P* value^c^
1	*GJB5*	rs12402440	107,166,694	G	T	20.91	4.03 × 10^−6^
3	*LOC100288428*	rs59756727	7,656,037	A	T	44.08	6.02 × 10^−7^
6	*SNRNP48*	rs7760212	8,242,302	A	G	32.02	7.47 × 10^−7^
13	EFNB2*	rs2391333	34,968,257	T	C	41.42	9.17 × 10^−8^
13	*MTIF3*	rs9512706	28,041,615	A	C	19.87	3.94 × 10^−6^
19	*ZNF787*	rs668056	56,595,733	T	C	28.74	2.22 × 10^−6^
22	*ISX*	rs5755279	35,186,350	G	A	23.91	3.21 × 10^−6^

The nearest gene to which the SNP is located.

**Figure 3:**
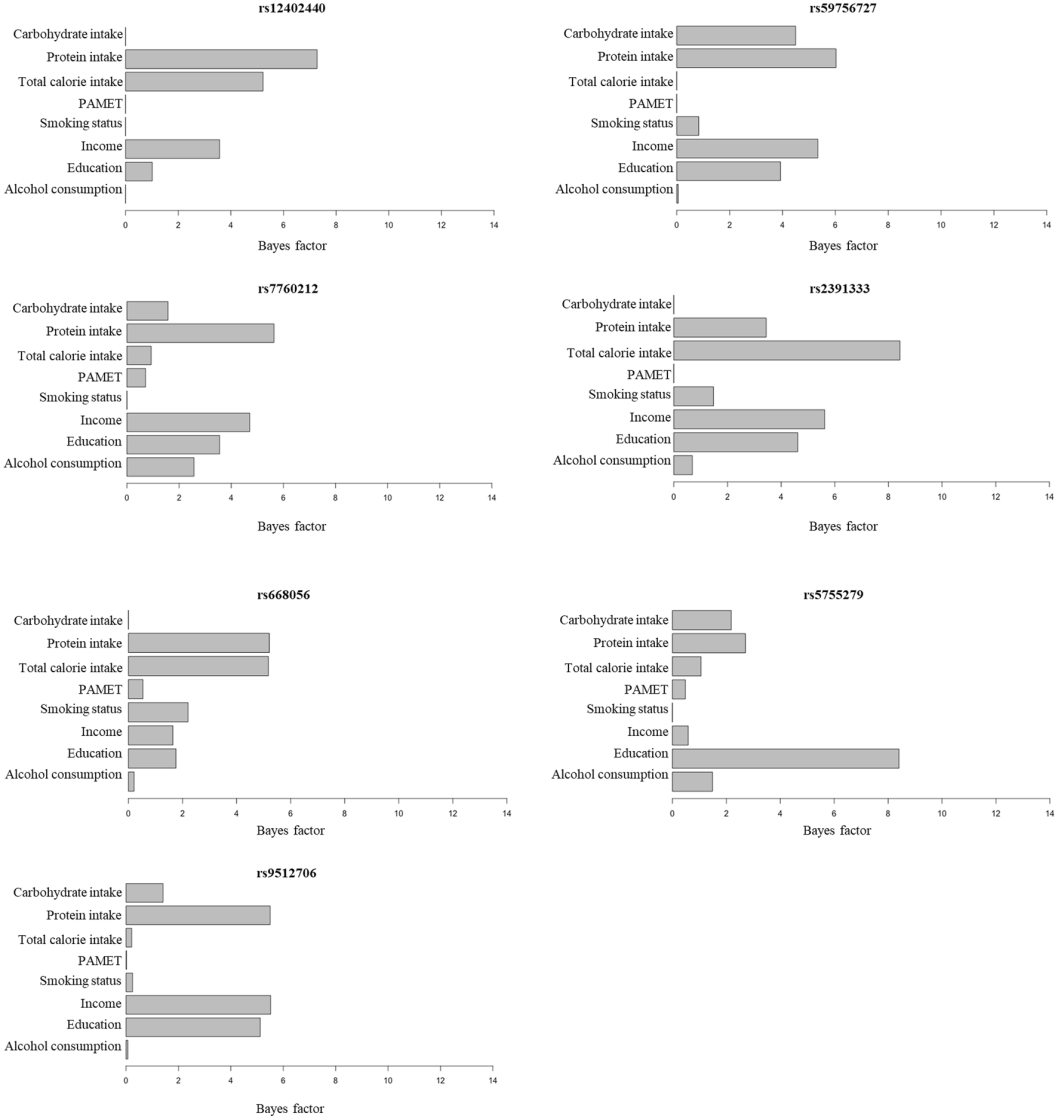
Interaction Bayes factor values of environmental factors for 7 potential single nucleotide polymorphisms (SNPs). Bayes Factor shows evidence of environmental factors that explain GEI at potential SNP.

For each potential genetic variant, we calculated the BF value of each environmental factor to examine the interaction reliability of the respective environmental factor on BMI, using the tool included in the Struct-LMM program^18^. For rs2391333, showing the most significant interaction (*P*-value = 9.17 × 10^−8^), the BF value of total calorie intake was the highest (BF = 8.43) among the 8 environmental factors, while the BF values of both carbohydrate intake and PAMET were the lowest (BF < 0.01 each, Fig. 3). When the BF values of the environmental factors were added from all the 7 potential SNPs, the BF value of PAMET was the lowest among the 8 environmental factors (Fig. 3, Supplementary Table 2). To confirm whether this was true for other SNPs as well, we recalculated the BF value of the 8 environmental factors from 66 independent lead SNPs that showed an interaction association with *P*-value < 5 × 10^−5^ (Supplementary Table 3). A BF value < 1 indicates that the environmental factors, through the GEI, does not improve the test power of Struct-LMM model. In another word, the environmental factor does not help in identifying genetic factors affecting BMI through the interaction. When the BF from all 66 SNPs were added, the BF value of PAMET was found to be the lowest compared to other environmental factors (BF_PAMET_ = 57.95). In addition, the average BF value of PAMET was < 1. (BF_PAMET_ average = 0.88, BF_Alcohol consumption_ average = 1.30, BF_Education_ average = 2.78, BF_Income_ average = 3.34, BF_Smoking status_ average = 1.17, BF_Total calorie intake_ average = 1.41, BF_Protein intake_ average = 2.87, and BF_Carbohydrate intake_ average = 1.50) (Supplementary Table 3). Therefore, we analyzed the GEI again by Struct-LMM, after excluding PAMET.

### Second analysis of gene–environment interaction using 7 environmental factors

**Figure 4:**
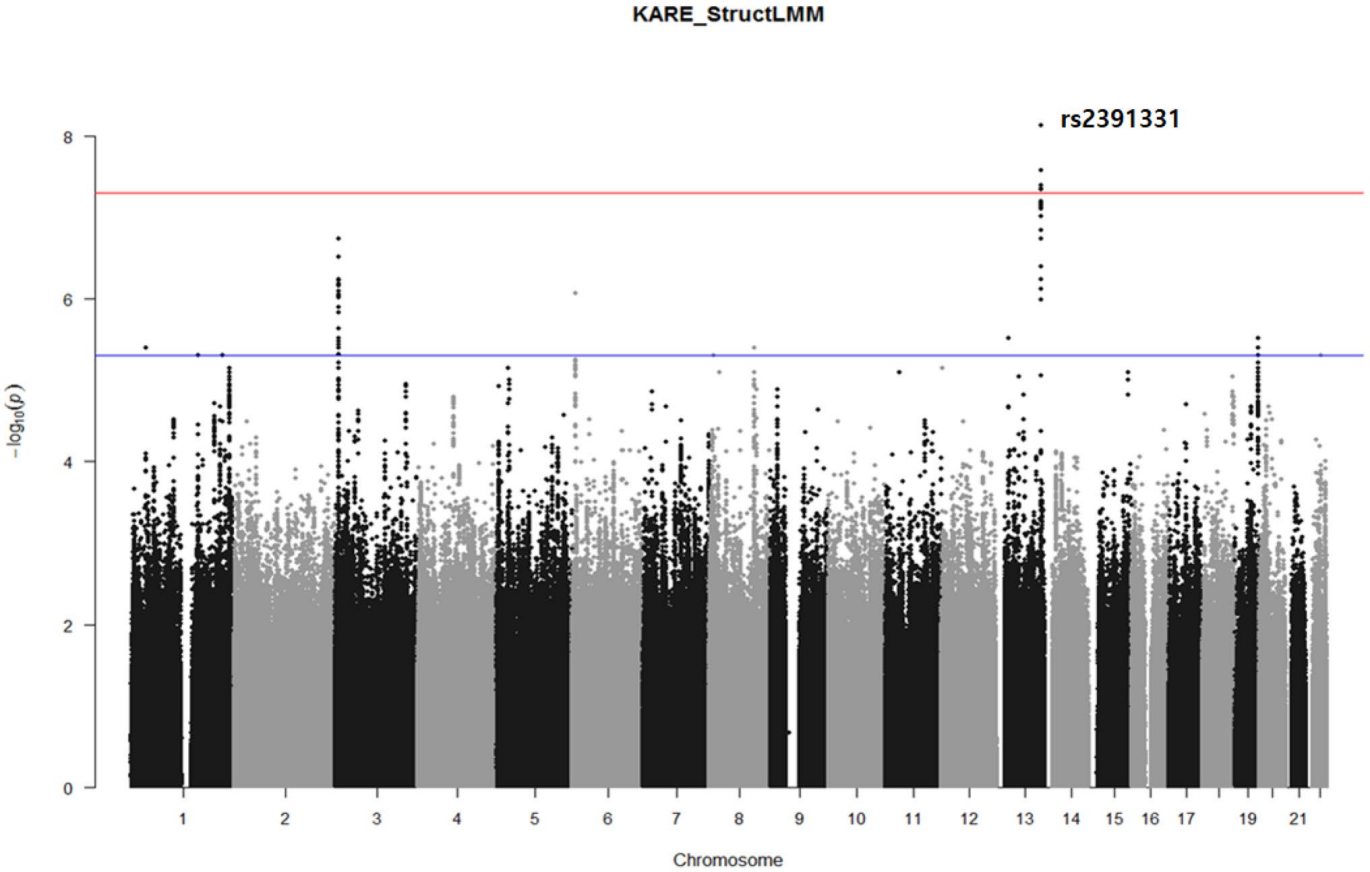
Manhattan plots for genome-wide interaction study using 7 environmental factors. The red line indicates the suggestive threshold (*P* value < 5.00 × 10^−6^). The blue line indicates the genome-wide significance threshold (*P* value < 5.00 × 10^−8^).

We performed the GEI test between the 5,908,111 SNPs and 7 environmental factors (without PAMET) on BMI, using Struct-LMM adjusted for age, sex, and recruitment area (Fig. 1). We discovered 11 independent potential genetic variants with *P*-value < 5 × 10^−6^ (Table 3, Fig. 4). A variant, rs2391331, located within the intron of *EFNB2* gene (Chr 13), showed a genome-wide significance (*P*-value = 7.27 × 10^−9^).

**Table 3:** Interactions between individual single nucleotide polymorphisms (SNPs) and 7 environmental factors. ^a^Chromosomal positions are based on the 1000 Genomes Project’s haplotype phase 1 in NCBI build 37 (hg19). ^b^MAF is minor allele frequency. ^c^The *P* value for the effects of interaction between genotypes and environmental factors on BMI were assessed by using a Struct-LMM with adjustment for age, sex, and recruitment area.

Chromosome	Nearest gene	SNP ID	Position^a^	Minor allele	Major allele	MAF^b^ (%)	*P* value^c^
1	*GJB5*	rs12402440	34,968,257	G	T	20.91	4.02 × 10^−6^
1	*C1orf110*	rs12405096	162,926,260	T	C	41.78	4.60 × 10^−6^
1	RAB3GAP2*	rs74565497	220,415,930	G	A	8.12	4.82 × 10^−6^
3	*LOC100288428*	rs59756727	8,242,302	A	T	44.08	1.81 × 10^−7^
6	*SNRNP48*	rs7760212	7,656,037	A	G	32.02	8.62 × 10^−7^
8	MSRA*	rs117301188	10,070,612	A	C	9.62	4.70 × 10^−6^
8	RSPO2*	rs7833349	108,966,199	T	C	24.70	3.81 × 10^−6^
13	*MTIF3*	rs9512706	28,041,615	A	C	19.87	2.91 × 10^−6^
13	EFNB2*	rs2391331	107,157,709	C	T	42.90	7.27 × 10^−9^
19	*ZNF787*	rs668056	56,595,733	T	C	28.74	2.87 × 10^−6^
22	*ISX*	rs5755279	35,186,350	G	A	23.91	4.65 × 10^−6^

The nearest gene to which the SNP is located.

We analyzed the interaction association of the other 6 potential variants identified from the analysis with the 8 environmental factors, including PAMET. While three SNPs (rs12402440, rs59756727, and rs9512706) had lower *P*-values in the interaction test involving 7 environmental factors, the other 3 SNPs (rs7760212, rs668056, and rs5755279) had higher *P*-values (Supplementary Table 4).

Since waist and hip circumferences were also used as indices for obesity, we analyzed the interaction of rs2391331 with 7 environmental factors on both these traits by Struct-LMM analysis. Both the correlation between BMI and waist circumference was 0.76 (*P* value < 5.00 × 10^−16^), and the correlation between BMI and hip circumference was also 0.76 (*P* value < 5.00 × 10^−16^) in this study population. Results showed that rs2391331 interacted with the 7 environmental factors on waist circumference (*P* value = 1.50 × 10^−5^) and hip circumference (*P* value = 6.00 × 10^−6^) (Supplementary Table 5).

The Struct-LMM analysis examined the interaction of a genetic variant with multiple environment factors as a whole. Hence, we investigated the interaction of individual environmental factors with rs2391331 using a fixed effect model of linear regression. As shown in Supplementary Table 6, rs2391331 showed interaction *P* value with protein intake (*P*-value = 1.64 × 10^−4^), income (*P* value = 3.62 × 10^−4^), total calorie intake (*P*-value = 6.27 × 10^−4^), alcohol consumption (*P*-value = 2.90 × 10^−3^), smoking status (*P*-value = 3.77 × 10^−3^), education (*P* value = 1.88 × 10^−2^), and carbohydrate intake (*P*-value = 5.28 × 10^−1^).

### Gene-based genome-wide association analysis

In order to validate the previous results of Struct-LMM and also identify the causative genes, we performed a gene-based genome-wide association analysis using the MAGMA^26^ tool through GWIS results calculated from Struct-LMM (Supplementary Fig. 3). SNPs were mapped to 17,535 protein-coding genes, making the genome-wide significance level as defined at *P*-value = 2.85 × 10^−6^ (0.05/17,535). Three genes of *EFNB2* (Chr 13), *DOCK4* (Chr 7), and *ZNF787* (Chr 19) met the genome-wide significance level, and the *P*-value of 3 genes were as follows: *EFNB2 P*-value = 5.03 × 10^−10^, *DOCK4 P*-value = 6.79 × 10^−7^, and *ZNF787 P*-value = 2.34 × 10^−6^ (Supplementary Table 7). The SNPs with the highest GEI *P*-value in the gene locus were as follows: [*DOCK4*—rs3801778 (GEI *P*-value = 8.90 × 10^−5^)], [*EFNB2*—rs2391331 (GEI *P*-value = 7.27 × 10^−9^)], and [*ZNF787*—rs642776 (GEI *P*-value = 3.00 × 10^−6^)]. We summarized the BF values of each environmental factor for rs3801778 and rs642776 in Supplementary Table 8. Further, we investigated the expression quantitative trait loci (eQTL) information using Genotype-Tissue Expression (GTEx) version 8^30^. We could find eQTLs of LD-linked proxy SNPs (r^2^ > 0.8) from rs2391331 and rs642776, and summarized the results in Supplementary Table 9. The proxy SNPs, rs10508174, rs11069646, rs7983579, and rs7327929 for rs2391331 are associated with *EFNB2* gene in the pituitary or testis. The proxy SNPs, rs7250351, rs1007851, rs6509982, rs493717, and rs35766803 for rs642776 are associated with *ZNF787* gene in fibroblast cells, esophagus, or skin, and the proxy SNPs, rs6509982 and rs35766803 for rs642776 are associated with *TMEM190* gene in the lung.

### BF of gene–environment associated variant

**Figure 5:**
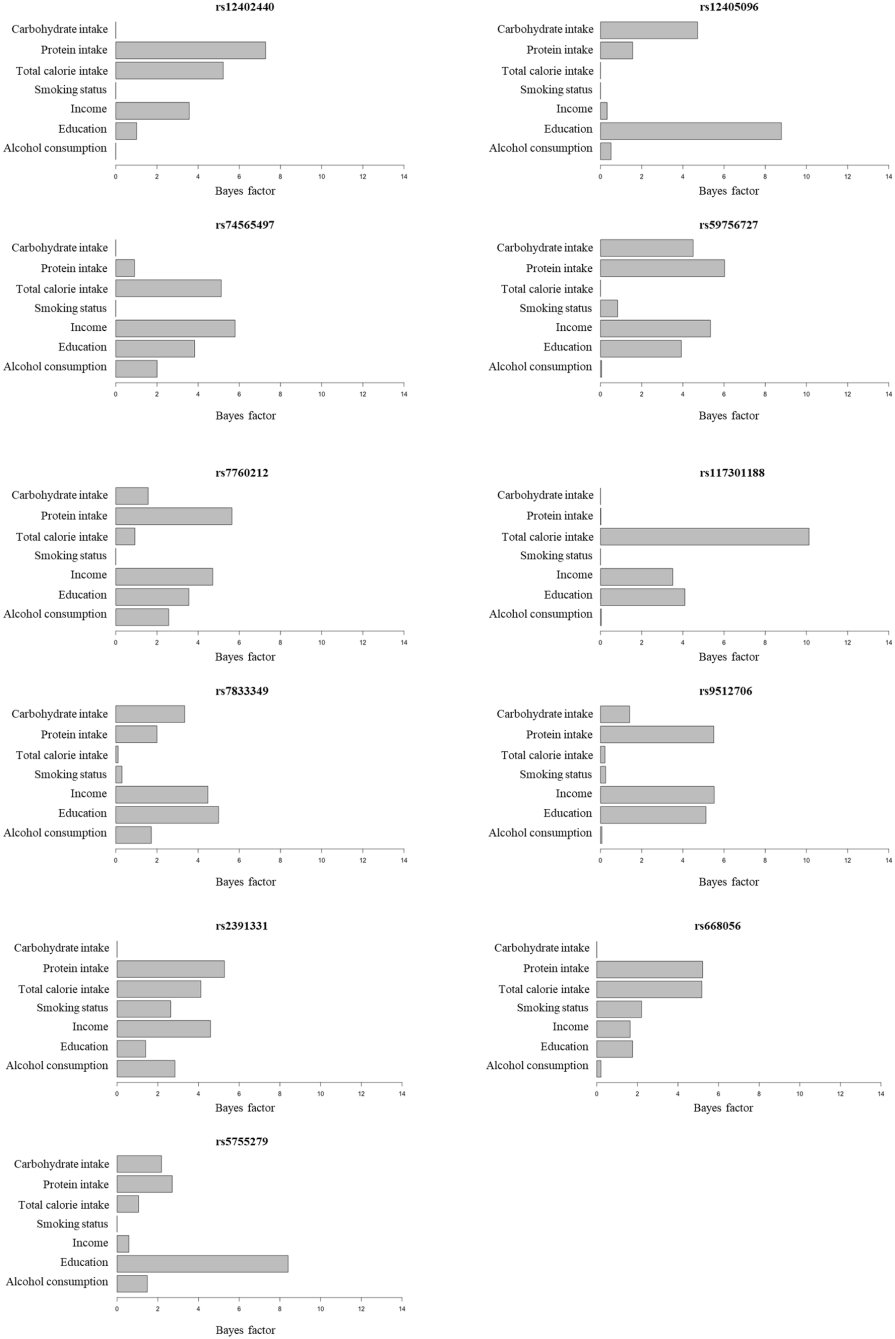
Interaction Bayes factor values of environmental factors for 11 potential single nucleotide polymorphisms (SNPs). Bayes Factor shows evidence of environmental factors that explain GEI at potential SNP.

The BF of environmental factors for the 11 potential SNPs were calculated (Fig. 5, Supplementary Table 10). For the most significant variant, rs2391331, all the environmental factors (except carbohydrate intake), showed BF value > 1, thus indicating the evidence of interaction. The interaction BF values of rs2391331 were as follows: protein intake (BF = 5.28), income (BF = 4.59), total calorie intake (BF = 4.12), alcohol consumption (BF = 2.85), smoking status (BF = 2.63), and education (BF = 1.41) (Supplementary Table 10). Additionally, we investigated the interaction of individual environmental factors with the 10 potential SNPs using a fixed effect model of linear regression (Supplementary Table 11–20). As a result, the most significant *P*-value (*P*-value = 7.65 × 10^−7^) was observed with total calorie intake interacted with rs11730118, which also showed the highest BF value (BF = 10.12) among the 11 potential SNPs of Struct-LMM analysis (Fig. 5, Supplementary Table 10, and Supplementary Table 16).

Box plots showed distribution of the in sample estimated allelic effect size on BMI considering environmental factors for 11 potential variants with GEI (n = 8155, unrelated individuals of Korean population) (Supplementary Fig. 2).

## Discussion

In this study, we performed an interaction test between multiple environmental factors and genetic variant, using Struct-LMM at a genome-wide level^18^. The identified genetic variant, rs2391331 was significantly associated with the interaction of 7 environmental factors in a Korean population cohort, KARE (*P*-value = 7.27 × 10^−9^). Of the 7 environmental factors, protein intake was the most influential environmental factor for rs2391331, and the least influential environmental factor was carbohydrate intake (Fig. 5).

A certain genetic variant may affect a trait through multiple environmental factors, as shown in *FTO* and *MC4R*^31–33^. The genetic variant of *FTO* showed interactions with diverse environmental exposures such as physical activity, diet, and alcohol consumption. The effect size of *FTO* variants on BMI was reduced by the increased physical activity but increased by the decreased physical activity^10^. Struct-LMM was modelled to identify such interactions with multiple environmental factors, which may not be identified otherwise. As shown in Supplementary Table 6, rs2391331 could not be identified as a genome-wide significant variant through the conventional fixed effect model approach, which used a single environmental factor. While protein intake, income, total calorie intake, alcohol consumption, and smoking status showed statistical significance after multiple correction, none of these environmental factors showed a genome-wide significance for the interaction with rs2391331 (*P*-value < 5.00 × 10^−8^). Therefore, unless the Struct-LMM model was applied, rs2391331 could not be identified through the genome-wide interaction study.

Another advantage of the Struct-LMM analysis is that it provides a rank about which environmental factor is more reliable for the interaction with the genetic variant, using BF values. As shown in Fig. 5 and Supplementary Table 10, the BF values for rs2391331 indicated that the ranking was in the order of protein intake (BF value = 5.28), income (BF = 4.59), total calorie intake (BF = 4.12), alcohol consumption (BF = 2.85), smoking status (BF = 2.63), education (BF = 1.41), and carbohydrate intake (BF < 0.01). When we investigated the interaction of individual environmental factors with rs2391331 using a fixed effect model of linear regression, the *P*-values of environmental factors were in the order of *P*-value, as follows: protein intake (*P*-value = 1.64 × 10^−4^), income (*P*-value = 3.62 × 10^−4^), total calorie intake (*P*-value = 6.27 × 10^−4^), alcohol consumption (*P*-value = 2.90 × 10^−3^), smoking status (*P*-value = 3.77 × 10^−3^), education (*P*-value = 1.88 × 10^−2^), and carbohydrate intake (*P*-value = 5.28 × 10^−1^) as shown in Supplementary Table 6. The order of *P*-value of each environmental factor was the same as the one of BF value obtained by Struct-LMM. In case of rs2391331, all 6 environmental factors (except carbohydrate intake) had a rather consistent effect on BMI through the interaction.

The genetic variant, rs2391331 was located in the first intron of *EFNB2* gene (Chr 13). EFNB2 is the ligand of erythropoietin-producing hepatocellular kinases (EPH), the largest family of receptor tyrosine kinases^34, 35^. We performed the gene-based genome-wide association analysis and verified that at the gene level *EFNB2* was also statistically significant for the interaction with the 7 environmental factors (Gene-set *P*-value = 5.03 × 10^−10^) (Supplementary Table 7). Further, we found that LD-linked proxy SNPs for rs2391331 were associated with *EFNB2* gene in the pituitary, or testis (Supplementary Table 9). *EFNB2* has been reported to be associated with schizophrenia and ankle injury through the genome-wide association study. Also, several studies have shown the association of this gene hypertension and type 2 diabetes; however, there have been no reports regarding the connection of *EFNB2* with obesity-related traits^36–38^. Recently, it was reported that smooth muscle-specific deletion of *EFNB2*, using SM22Δ-Cre, resulted in lower body weight, reduced vascular smooth muscle cell proliferation, wall, and enlarged arterial diameter^39^. More recently, it has been reported that the expression of *EFNB2* is enriched in proopiomelanocortin (POMC) neurons, a major regulator of energy balance and glucose homeostasis and the loss of *EFNB2* in POMC-expressing progenitors mildly impairs gluconeogenesis and food intake in mice^40^. We do not know yet whether how this report would be related to the findings in this study. Further studies are required to investigate the functional analysis of *EFNB2* related to obesity.

Additionally, we found 4 publications related to the GEI on BMI in which studies 96 SNPs were reported^18,41–43^. We tested the 96 SNPs for replication using the Struct-LMM in this study population, however we could not find any significant interaction under the multiple correction criteria (*P*-value < 0.05 / 96) (Supplementary Table 21). We may provide the reasons for the failure of replication as follows. First, our sample size (N = 8,155) may not be enough for replication. It is generally accepted that for GEI studies, the bigger sample size is recommended than the size for GWAS^2^. Second, the ethnicity of our sample (East Asian population) is different from the ethnicity of previously mentioned 4 studies (3 studies in European population^18,41,43^; 1 study in African and Hispanic population^42^). The effects of environmental factors are supposed to be discordant between different ethnical backgrounds, making it hard to produce a replication. Third, there may be a limitation in obtaining the precise environmental data. The environmental data acquired from self-reported questionnaire (i.e., dietary intakes, alcohol, smoking, sociodemographic factors, and physical activities) may be prone to responder bias^9^.

There are several limitations to our study. First, as mentioned above, we analyzed the GEI in obesity with a small sample size, which can affect the statistical power and lead to imprecise or incorrect estimates of the magnitude of observed effects^44^. In addition, the number of environmental factors investigated was small. While Struct-LMM analysis has good detection power, even if more than 10 environmental factors are analyzed, a smaller number of environmental factors reduces the detection power of this method^18^. Lastly, the results of rs2391331 were not validated in other cohorts or ethnicities. Although it is not easy to find cohorts with all the diverse environmental factors similar to this study, replication of the results in other cohorts is warranted.

In conclusion, we performed multiple environments-gene interaction analysis to identify potential SNPs of BMI in a Korean cohort. A genome-wide significant interaction of rs2391331, located in the *EFNB2* locus, was identified and the interaction on BMI was influenced by 6 environmental factors, namely protein intake, income, total calorie intake, alcohol consumption, smoking status, and education.

## Supplementary Information

Supplementary Information 1.

Supplementary Information 2.
