# Supplementary material for: Identification of genetic loci affecting body mass index through interaction with multiple environmental factors using structured linear mixed model
Source: Sci Rep. 2021 Mar 2;11:5001. doi: 10.1038/s41598-021-83684-1 (PMC7925554; doi:10.1038/s41598-021-83684-1)
Supplement: Supplementary file 2 — Supplementary Information 2. [file 41598_2021_83684_MOESM2_ESM.pdf]

Identification of genetic loci affecting body mass index through interaction with multiple environmental factors using structured linear mixed model

Hae-Un Jung[1], Won Jun Lee[1], Tae-Woong Ha[1], Ji-One Kang[2], Jihye Kim[3], Mi Kyung Kim[3,4], Sungho Won[5], Taesung Park[6], Ji Eun Lim[2*] & Bermseok Oh[2*]

[1]Department of Biomedical Science, School of Medicine, Kyung Hee University, Seoul, South Korea

[2]Department of Biochemistry and Molecular Biology, School of Medicine, Kyung Hee University, Seoul, South Korea

[3]Department of Preventive Medicine, College of Medicine, Hanyang University, Seoul, South Korea

[4]Department of Public Health Science, College of Medicine, Hanyang University, Seoul, South Korea

[5]Department of Public Health Science, Seoul National University, Seoul, South Korea

[6]Department of Statistics, Seoul National University, Seoul, South Korea

*Corresponding author

Bermseok Oh

Department of Biochemistry and Molecular Biology, School of Medicine, Kyung Hee University, Seoul, South Korea.

Phone: +82 2-961-0617

E-mail: ohbs@khu.ac.kr

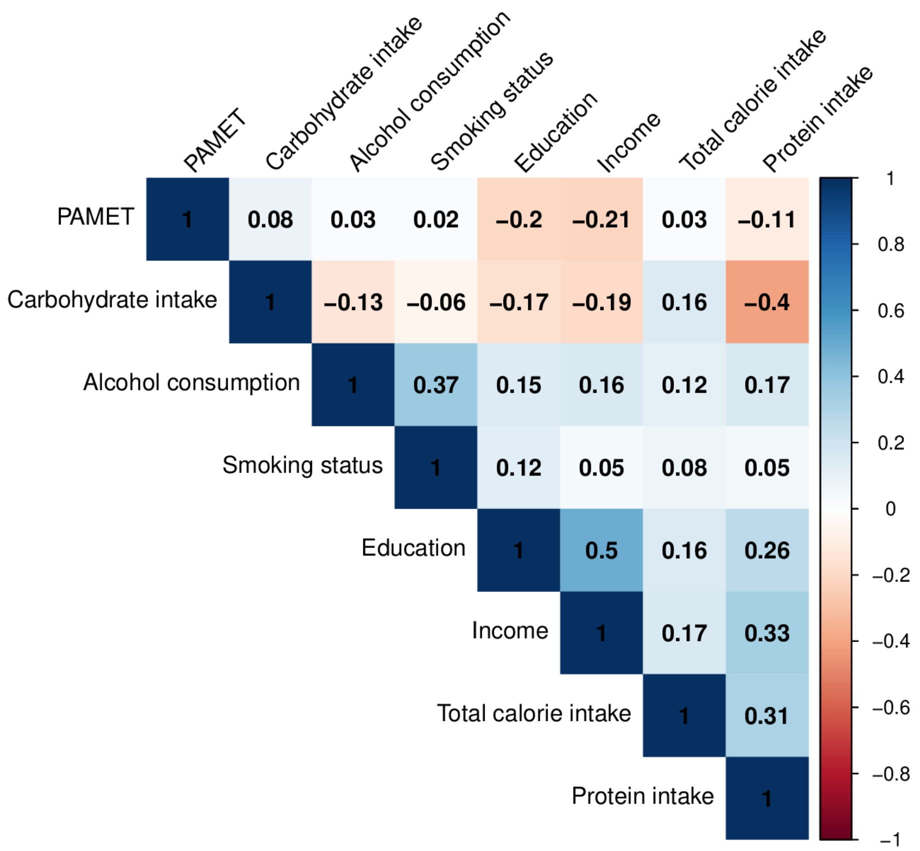

Supplementary Figure 1. Shown are the correlation coefficients between pairs of environmental factors, considering 8 body mass index (BMI)-related factors.

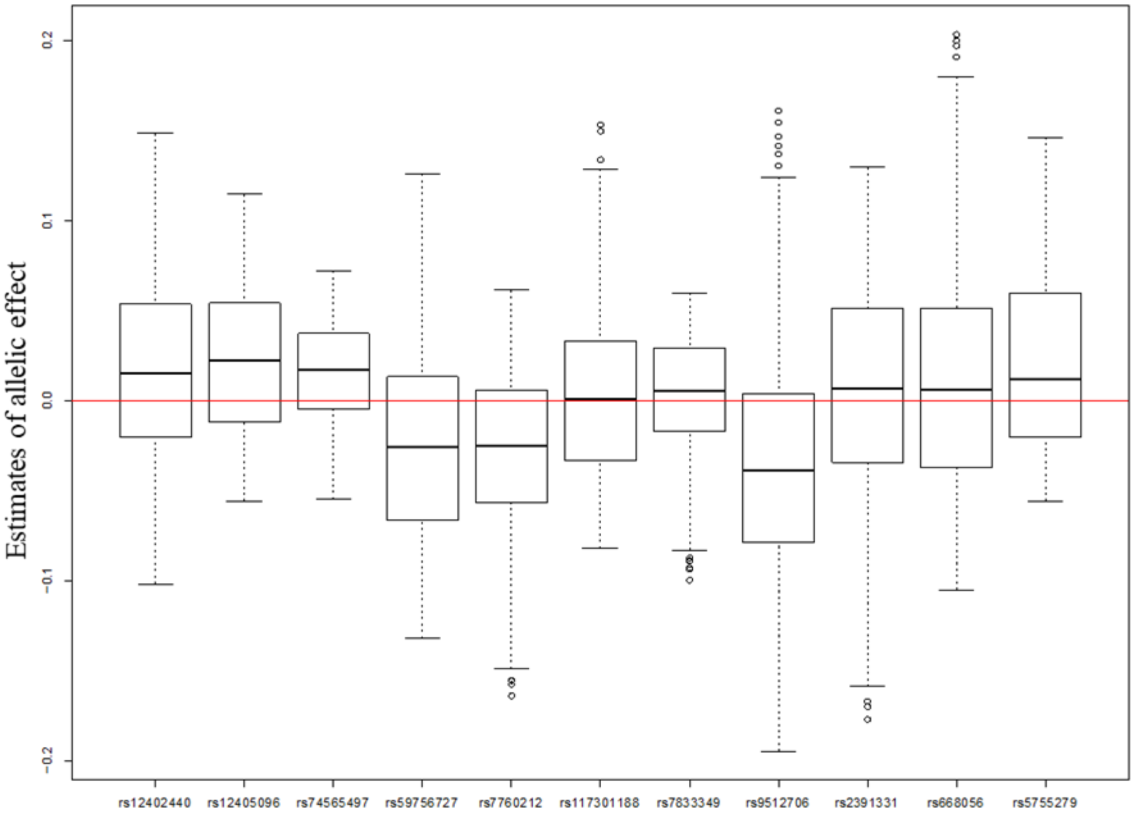

Supplementary Figure 2. Distribution of allelic effect in population considering both the allelic genetic effect and the interaction effect for 11 potential single nucleotide polymorphisms (SNPs).

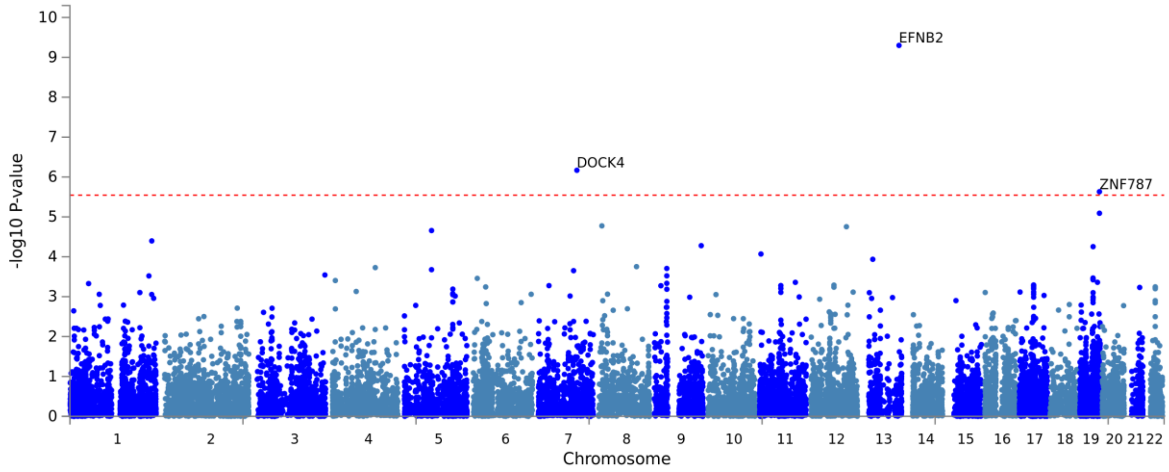

Supplementary Figure 3. Manhattan plots for gene-based genome-wide association analysis using MAGMA. The red line indicates the genome-wide significance threshold (*P*-value < 2.85 × 10^{-6}).
